# Supplementary material for: The Role of Honey Bee Derived Aliphatic Esters in the Host-Finding Behavior of Varroa destructor
Source: Insects. 2022 Dec 25;14(1):24. doi: 10.3390/insects14010024 (PMC9863403; doi:10.3390/insects14010024)
Supplement: Supplementary file 1 [file insects-14-00024-s001.zip › insects-2047733-supplementary.pdf]

Table S1. Qualification and quantification of volatiles on the body surface of honeybee larvae by GC-MS.

Peak No.	Retention time (min)	Proposed identity	Kovats Retention Index	Formula	Amount (ng/bee)							
					Am drone (n=26)		Am worker (n=22)		Ac drone (n=23)		Ac worker (n=19)	
					Mean	SD	Mean	SD	Mean	SD	Mean	SD
1	3.827	Toluene	755	C7H8	-	-	0.357	0.620	-	-	1.539	2.706
2	4.525	Octane	800	C8H18	2.467	1.855	1.049	1.133	-	-	-	-
3	5.057	Propanoic acid, 2,2-dimethyl-	864	C8H9NO2	-	-	-	-	0.213	0.689	-	-
4	5.118	Heptane, 2,4-dimethyl-	797	C9H20	0.657	0.858	1.419	0.949	-	-	0.552	0.673
5	6.475	Octane, 4-methyl-	866	C9H20	0.228	0.323	0.469	0.331	-	-	0.081	0.245
6	6.549	Unknown			-	-	-	-	-	-	1.403	3.362
7	7.967	Nonane	900	C9H20	0.175	0.619	-	-	-	-	-	-
8	8.724	Unknown			3.053	3.738	2.722	3.031	1.291	2.674	5.035	2.760
9	10.156	2(3H)-Furanone, dihydro-5-methyl-	921	C5H8O2	-	-	0.078	0.367	-	-	-	-
10	11.018	Unknown			0.431	0.759	-	-	-	-	0.514	1.053
11	11.121	Unknown			0.838	1.319	-	-	-	-	-	-
12	12.584	Unknown			0.540	0.646	-	-	-	-	-	-
13	12.788	Bicyclo[3.1.1]heptane, 6,6-dimethyl-2-methylene-, (1S)-	972	C10H16	-	-	0.147	0.689	-	-	-	-
14	13.169	Hexanoic acid	983	C6H12O2	-	-	0.339	1.176	0.870	1.255	-	-
15	13.401	Unknown			-	-	0.502	0.869	-	-	-	-
16	14.302	Unknown			-	-	3.565	2.989	-	-	-	-
17	14.767	D-Limonene	1024	C10H16	-	-	2.120	1.155	-	-	1.207	1.081
18	15.055	1-Hexanol, 2-ethyl-	1012	C8H18O	-	-	3.098	1.197	-	-	2.322	1.957
19	15.548	trans-β-Ocimene	1029	C10H16	-	-	0.215	0.632	-	-	-	-
20	15.743	Unknown			0.279	0.362	-	-	-	-	-	-
21	16.060	β-Ocimene	1038	C10H16	-	-	14.914	23.274	-	-	-	-
22	16.673	Dodecane	1200	C12H26	0.898	1.338	1.779	1.803	-	-	1.493	1.314
23	16.943	2,6-Dimethyldecane	1119	C12H26	0.270	0.388	0.359	0.488	-	-	-	-
24	17.123	Unknown			0.440	1.347	0.283	0.877	-	-	-	-

25	17.179	Unknown			0.754	0.829	0.090	0.250	-	-	-	-
26	17.807	Unknown			-	-	0.301	0.657	-	-	-	-
27	17.973	trans-Linalool oxide (furanoid)	1060	C10H18O2	1.167	2.287	0.265	1.106	-	-	-	-
28	18.206	Benzoic acid, methyl ester	1062	C8H8O2	6.220	4.316	0.565	1.304	-	-	-	-
29	18.644	Linalool	1080	C10H18O	0.289	0.714	16.666	14.317	-	-	-	-
30	18.870	Nonanal	1073	C9H18O	-	-	-	-	2.325	3.887	-	-
31	19.206	Benzene, 1,2,4,5-tetramethyl-	1101	C10H14	0.300	0.431	0.040	0.189	1.368	1.554	-	-
32	19.695	1,3,8-p-Menthatriene	1119	C10H14	-	-	0.121	0.394	-	-	-	-
33	20.104	2,6-Dimethyl-1,3,5,7-octatetraene, E,E-	1130	C10H14	-	-	1.346	2.714	-	-	-	-
34	20.699	2,4,6-Octatriene, 2,6-dimethyl-, (E,Z)-	1131	C10H16	-	-	1.121	2.161	-	-	-	-
35	23.239	L-α-Terpineol / α-Terpineol	1198	C10H18O	-	-	1.776	1.846	-	-	-	-
36	23.380	Methyl salicylate	1176	C8H8O3	-	-	-	-	5.161	4.468	0.144	0.437
37	23.427	Octanoic acid	1158	C8H16O2	0.940	1.934	-	-	-	-	-	-
38	24.109	1,4:3,6-Dianhydro-.alpha.-d-glucopyranose	1199	C6H8O4	-	-	-	-	0.335	0.821	-	-
39	25.052	Unknown			0.055	0.280	-	-	-	-	-	-
40	26.686	Unknown			-	-	-	-	-	-	0.362	0.890
41	29.227	Nonanoic acid	1263	C9H18O2	0.110	0.428	-	-	-	-	-	-
42	30.125	Unknown			-	-	0.591	2.773	-	-	-	-
43	30.674	Tridecane	1300	C13H28	2.846	1.860	0.064	0.298	-	-	-	-
44	32.477	Methyl anthranilate	1320	C8H9NO2	0.480	2.101	-	-	-	-	-	-
45	32.645	Unknown			0.474	1.727	-	-	0.146	0.700	-	-
46	34.559	2-Decenoic acid, methyl ester	1362	C11H20O2	-	-	-	-	1.457	2.423	-	-
47	36.726	2,4,7,9-Tetramethyl-5-decyne-4,7-diol	1407	C14H26O2	-	-	-	-	0.352	0.679	-	-
48	37.903	Ethyl trans-2-decenoate	1166	C12H22O2	-	-	-	-	4.848	5.931	0.663	1.221
49	40.023	Pentadecane	1500	C15H32	6.090	4.164	1.927	1.223	-	-	-	-
50	40.305	2,4-Di-tert-butylphenol	1519	C14H22O	-	-	-	-	7.130	5.797	-	-
51	46.233	2-Pentadecanone	1682	C15H30O	-	-	-	-	0.479	0.653	-	-
52	46.345	Heptadecane	1700	C17H36	3.961	2.716	1.044	0.949	-	-	-	-
53	48.904	Tetradecanoic acid, ethyl ester (Ethyl	1778	C16H32O2	10.38	11.45	2.491	1.248	4.543	5.950	2.119	0.818

		myristate)			9	8						
54	51.641	Nonadecane	1900	C19H40	1.204	1.035	0.033	0.155	-	-	-	-
55	52.248	Hexadecanoic acid, methyl ester	1908	C17H34O2	1.877	3.965	0.408	1.245	1.865	3.691	2.130	0.909
		(Methyl palmitate)										
56	53.179	n-Hexadecanoic acid	1941	C16H32O2	0.186	0.414	-	-	0.409	0.974	-	-
57	53.235	Ethyl 9-hexadecenoate	1955	C18H34O2	0.157	0.338	-	-	-	-	-	-
58	53.901	Hexadecanoic acid, ethyl ester	1976	C18H36O2	13.87	16.64	1.530	0.917	9.736	9.346	5.695	2.259
		(Ethyl palmitate)			4							
59	56.301	9-Octadecanoic acid , methyl ester,(E)-	2098	C19H38O2	0.403	0.937	-	-	0.198	0.407	0.079	0.237
60	56.314	Heneicosane	2100	C21H44	0.075	0.185	-	-	-	-	-	-
61	57.671	Ethyl Oleate	2171	C20H38O2	3.155	6.171	0.368	0.500	1.751	1.953	1.203	0.755
62	58.221	Octadecanoic acid, ethyl ester	2175	C20H40O2	0.290	0.793	-	-	0.034	0.095	-	-
63	60.042	Tricosane	2300	C23H48	0.176	0.371	-	-	-	-	-	-

Am: Apis mellifera; Ac: Apis cerana; "-": not detected. Compounds that cannot be properly identified are marked as "unknown".
